# Renal Transplant Immunosuppression Impairs Natural Killer Cell Function *In Vitro* and *In Vivo*

**DOI:** 10.1371/journal.pone.0013294

**Published:** 2010-10-12

**Authors:** Olivier Morteau, Samkeliso Blundell, Aron Chakera, Sophia Bennett, Charita M. Christou, Philip D. Mason, Richard J. Cornall, Christopher A. O'Callaghan

**Affiliations:** 1 Nuffield Department of Clinical Medicine, University of Oxford, Oxford, United Kingdom; 2 Oxford Kidney Unit, Churchill Hospital, Oxford, United Kingdom; Louisiana State University, United States of America

## Abstract

**Background:**

Despite an increasing awareness of the importance of innate immunity, the roles of natural killer (NK) cells in transplant rejection and antiviral and cancer immunity during immunosuppression have not been clearly defined.

**Methods:**

To address this issue we have developed a quantitative assay of NK cell function that can be used on clinical samples and have studied the influence of immunosuppression on NK cell function. NK cell degranulation and intracellular interferon (IFN)-γ production were determined by flow cytometry of peripheral blood samples.

**Results:**

Overnight *ex vivo* treatment of peripheral blood cells from healthy controls with ciclosporin or tacrolimus inhibited NK cell degranulation and IFN-γ production in a dose-dependent manner. A similar impairment of function was seen in NK cells from patients treated *in vivo* with calcineurin inhibitors. In the early post-transplant period, there was a variable reduction of NK cell counts after treatment with alemtuzumab and basiliximab.

**Conclusions:**

The functional inhibition of NK cells in early transplant patients coincides with the period of maximum susceptibility to viral infections. The ability to assay NK cell function in clinical samples allows assessment of the impact of immunosuppression on these effector cells. This information may be helpful in guiding the titration of immunosuppression in the clinical setting.

## Introduction

Natural killer (NK) cells have potent effector functions and play a key role in a range of immune responses, including those against pathogens and cancers [1]; however, their role in transplantation and response to transplant immunosuppression are not clearly defined. NK cells provide innate immunity against abnormal cells, where their activation depends on the integration of signals arising from activating and inhibitory receptors on their cell surface [2]. The inhibitory receptors include the CD94/NKG2A/B heterodimers, which we identified as receptors for human leukocyte antigen (HLA)-E, and killer inhibitory receptors (KIR) which interact with major histocompatibility complex (MHC) class I molecules [3], [4], [5]. Activating receptors include NKG2D, which interacts with a range of ligands including the highly polymorphic MICA and MICB molecules, the natural cytotoxicity receptors such as NKp46 and the KIR-like receptors, whose ligand repertoires are not fully characterized, but include HLA molecules [6], [7], [8]. An important feature of the NK cellular immune response is that NK cells do not require prior sensitisation in order to exert their effector function [1].

NK cells have two key effector functions, which are the cytotoxic lysis of target cells and the release of inflammatory cytokines that amplify the immune response, including interferon (IFN)-γ [1]. Around 90% of human peripheral blood NK cells are characterized by a CD56^dim^ phenotype and display a high level of cytotoxicity, while the remaining 10% are CD56^bright^ and display greater cytokine secretion [9], [10]. The subsets are believed to represent sequential stages of maturation, in which the cytokine-secreting CD56^bright^ cells give rise to more differentiated CD56^dim^ killer cells [9].

NK cells are important in the early stages of viral infections and NK cell deficiency predisposes to virus infections, in particular from herpes viruses [11], [12]. The role of NK cells in virus infection is especially important when adaptive immunity is not fully active and this is analogous to the situation with transplant immunosuppression [11]. Human NK cells can kill virus-infected cells, including those infected with cytomegalovirus, and cytomegalovirus encodes molecules that help it to evade NK cells [1], [13]. Ligands for human NKG2D are elevated on cytomegalovirus-infected cells and this receptor has been implicated in protection from cytomegalovirus infection in human transplant recipients [14].

It has been reported that human NK cells can mediate rejection of both allogeneic bone marrow and xenogeneic solid organ grafts [15], [16], [17], [18], [19]. Moreover, expression of the NK cell activating receptor NKG2D is increased with acute and chronic nephropathy after human kidney transplantation and may be a marker of acute and chronic transplant rejection [20]. Alloreactivity of NK cells can also promote allograft tolerance in animal models [21], [22], [23]. Despite the potential importance of NK cells in the context of transplantation, the impact of immunosuppressive strategies on NK cell numbers and functions is poorly understood. A number of *in vitro* studies of the actions of ciclosporin and tacrolimus on NK cell functions have produced contradictory observations ranging from inhibition to stimulation [24], [25], [26], [27], [28], [29].

In this study we show that NK cell function and numbers are reduced in transplant recipients, particularly at early time-points when there is the highest susceptibility to opportunistic viral infections. Accurate and simple assessment of NK cell function will be useful in developing algorithms to help clinicians to decide on the correct level of immunosuppression and to identify patients with the highest risk of infective complications.

## Results

### Refinement and validation of an assay of NK cell function

To measure NK cell function *ex vivo*, we first developed an assay of cytotoxic degranulation and cytokine production that was sensitive and reproducible using peripheral blood samples. Killing of target cells by NK cells results in cytotoxic degranulation with exposure of the inner lysosomal membrane protein CD107a at the NK cell surface, which can be detected by flow cytometry. Cytokine secretion by NK cells can be assessed simultaneously by flow cytometric evaluation of IFN-γ production. To measure these functions we exposed NK cells to known targets, which were either HLA class I-deficient Daudi cells transfected with MICA, a ligand for the activating receptor NKG2D (D860 cells), or untransfected K562 cells. Overnight rested peripheral blood mononuclear cells (PBMCs) (2.5×10^5^/well) were incubated in the presence or absence of target cells (1.25×10^6^/well). Following the incubation period, the cells were harvested, washed and stained with anti-human CD3, CD56 and CD16 antibodies for flow cytometry. For intracellular IFN-γ detection, cells were permeabilized and stained for IFN-γ. NK cells were identified by gating on the lymphocyte population and then on CD3^−^ CD56^+^ population. NK cell degranulation was expressed as the percentage of NK cells that were CD107a^+^ and cytokine secretion was expressed as the percentage of NK cells that were IFN-γ^+^.

**Figure 1:**
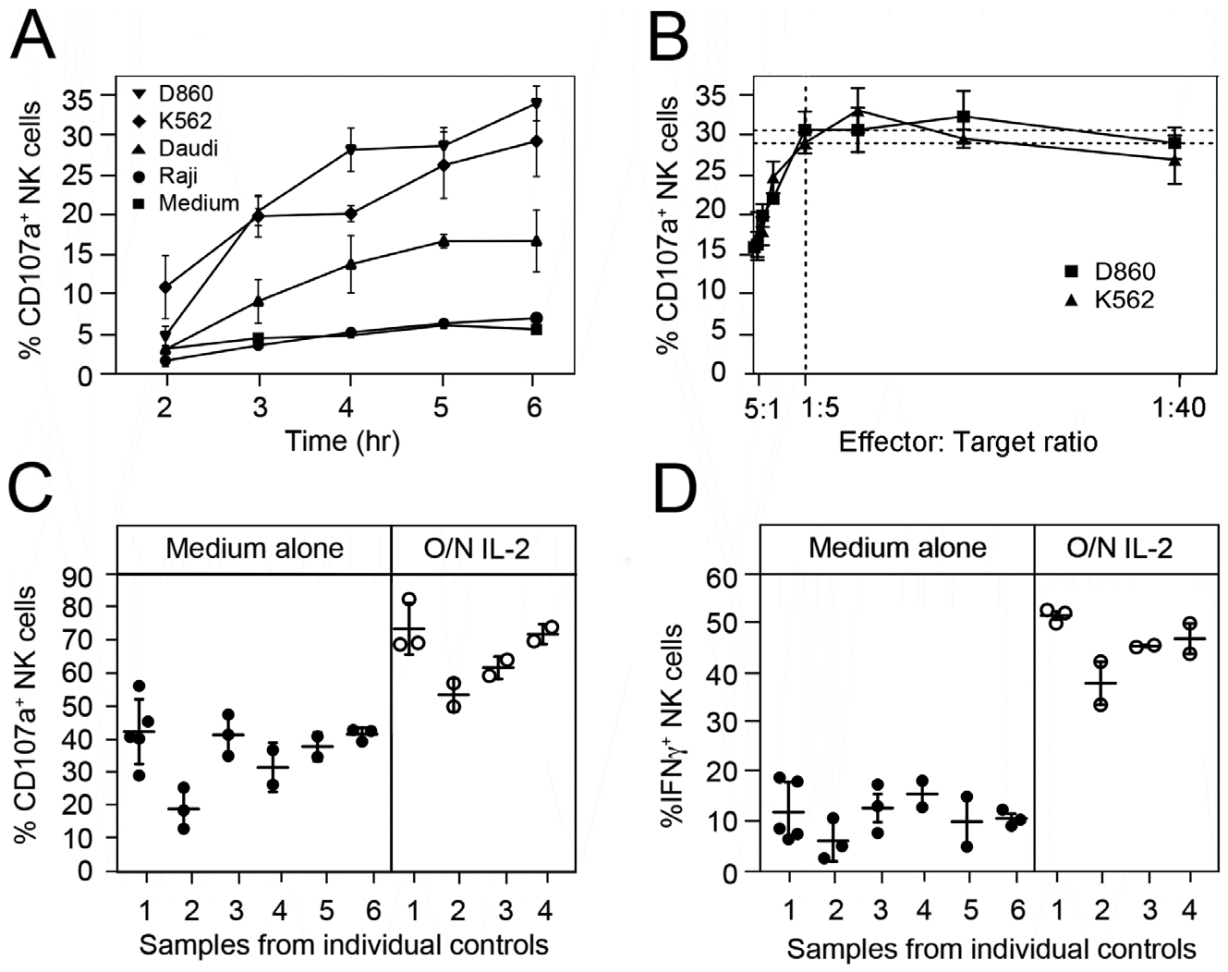
Development of an assay of NK cell function. (**A**) NK cell degranulation over time during incubation with different target cell lines at an effector:target ratio of 1∶5. The term ‘effector’ refers to the total number of peripheral blood mononuclear cells in the assay. **(B**) NK cell degranulation after 6 hr at effector:target ratios ranging from 5∶1 to 1∶40. (**C and D**). Reproducibility of the NK cell assay on different samples taken from individual healthy controls over several weeks (1–6) with a minimum interval of two weeks (individual circles). Closed and open circles on the left and right of each panel indicate values obtained when cells were incubated overnight prior to the assay in medium alone or medium plus IL-2 respectively. The data demonstrate the extent to which the assay is reproducible over time within an individual using a 6 hour incubation with K562 target cells and a 1∶5 effector:target ratio. Error bars represent standard deviations in all graphs in this figure.

Pilot studies of the assay using NK cells from healthy controls showed that there was a time-dependent increase in NK cell degranulation in response to the target cells with a near-maximal response seen at 6 hours (Figure 1A). Further experiments showed that responses were similar at 6 and 12 hours when both degranulation and intracellular IFN-γ production were assessed (data not shown); therefore the 6 hour incubation was used in future studies. We were also able to show that the proportion of NK cells that degranulated was not increased by increasing the number of target cells beyond an effector:target (E:T) cell ratios of 1∶5 (Figure 1B). The term ‘effector’ here refers to the total number of peripheral blood mononuclear cells added to the assay, but only the NK cells in this population were assayed. The flow cytometric assay excludes the analysis of CD3^+^ cells, but there was no significant degranulation of CD3^+^ cells in the presence of K562 or D860 target cells (0.13±0.003% and 0.43±0.02% respectively) and negligible change in the proportion of CD3^+^ cells secreting IFN-γ (0.003±0.002% vs. 0.02±0.002%). There was little difference in the efficiency of D860 and K562 target cells, so K562 cells were used in subsequent experiments. On the basis of these preliminary experiments, subsequent NK cell assays used a five-fold excess of target cells and incubation period of 6 hours. When absolute NK cell numbers were determined, no correlation was found between the ratio of NK:target cells in the assay results for 27 individuals (Figure S1); this ratio was generally of the order of 1∶100, with the smallest excess of target cells being a ratio of 1∶53. With an E:T ratio of 1∶5 and a 6 hour incubation with K562 cells, the test was sensitive and reproducible. The test remained reproducible for different individuals tested at different times over a period of several weeks (Figure 1C and 1D), with a mean coefficient of variation of 14.1% for degranulation and 28.5% for interferon gamma production. This is not the coefficient of variation of the assay itself, but rather of the assay result over time in any given individual. Overnight pre-activation of the NK cells with interleukin-2 (IL-2) increased the level of degranulation and the generation of IFN-γ, without altering the reproducibility of the assay (Figure 1C and 1D).

### Calcineurin inhibitors reduce NK cell degranulation and IFN-γ production *in vitro*

**Figure 2:**
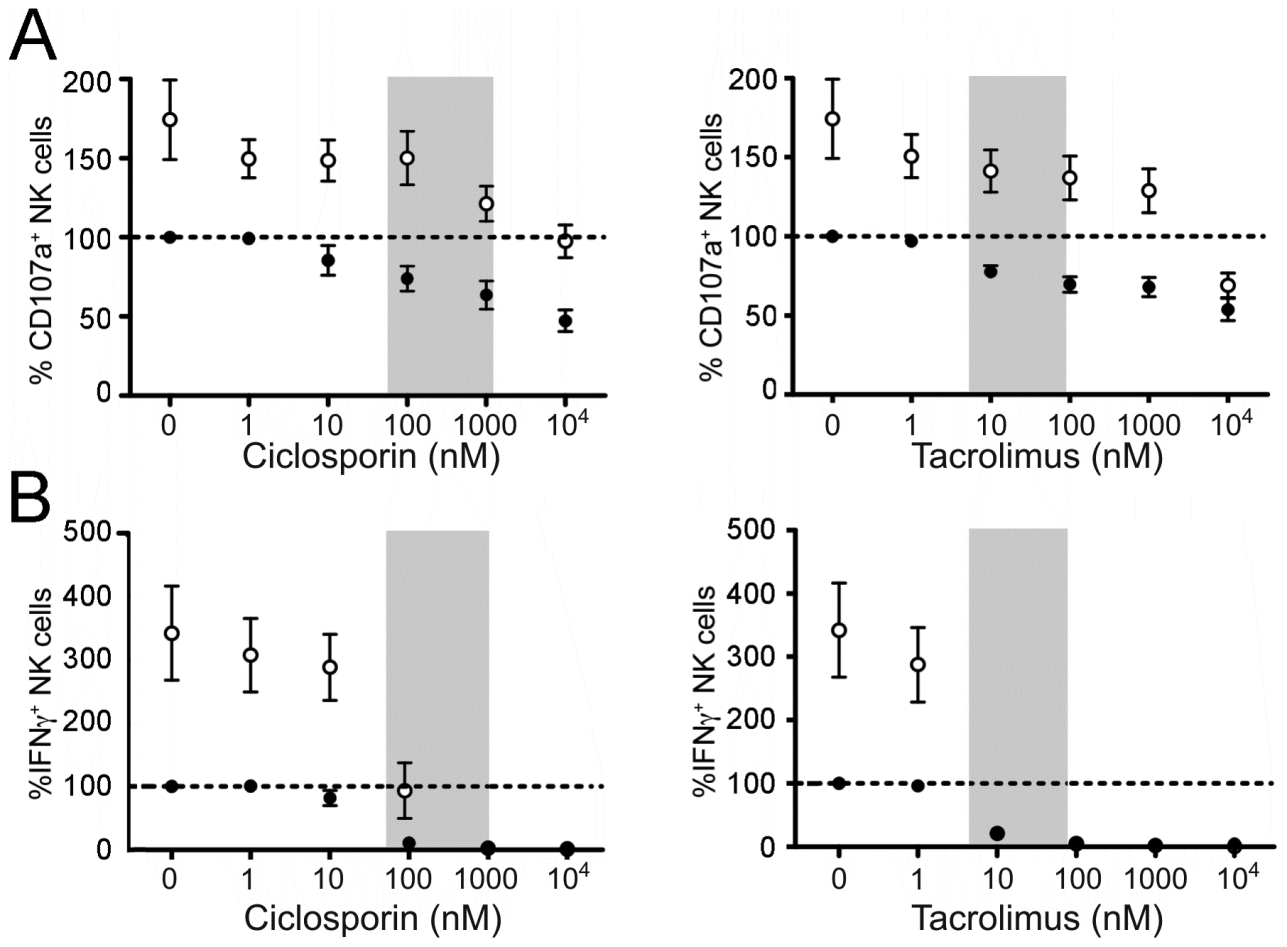
*Ex vivo* administration of ciclosporin or tacrolimus inhibits NK cell function. (**A**) PBMCs from healthy subjects (n = 4–8 for each data point) were incubated separately overnight with different concentrations of ciclosporin or tacrolimus with (open circles) or without (black circles) IL-2. NK cell degranulation was assessed the following day by incubation with K562 target cells for 6 hrs at a 1∶5 E:T ratio. The values for CD107a^+^ NK cells are expressed as a percentage of the basal level of CD107a^+^ surface expression in the assay for that individual in the absence of both the drugs and IL-2. The shaded area represents the range of drug levels encountered in clinical practice with renal transplantation. (**B**) IFN-γ production was evaluated by flow cytometry in the same assays as used in (A). The values of IFN-γ^+^ NK cells are expressed as a percentage of the basal level of IFN-γ^+^ expression for that individual in the assay in the absence of both the drugs and IL-2. Values are mean ± standard deviation. The data demonstrate that both ciclosporin and tacrolimus impair NK cell degranulation and IFN-γ production at levels corresponding to those used in clinical practice. In both A and B there was as strongly significant trend of inhibition (see Tables S1 and S2 for statistical analysis.).

Since the calcineurin inhibitors ciclosporin and tacrolimus are the mainstay of most immunosuppression regimens in renal transplantation, we studied their effects on NK cell function using this assay. Overnight treatment of cells from healthy donors with ciclosporin or tacrolimus caused a dose-dependent inhibition of NK cell degranulation and IFN-γ production in response to target cells (Figure 2A and 2B). A strong statistically significant inhibitory trend was seen with both drugs on degranulation and IFN-γ production (Tables S1 and S2.) At therapeutic trough levels of tacrolimus (6–20 nM or 5–15 ng/ml) and ciclosporin (60–250 nM or 75–300 ng/ml) there was a greater than 20% reduction in degranulation and a greater than 90% reduction in IFN-γ production. More substantial changes were seen in the range corresponding to the peak levels that can arise following therapeutic dosing with tacrolimus (40–90 nM or 30–70 ng/ml) or ciclosporin (800–1200 nM or 1000–1500 ng/ml). Overnight treatment of the NK cells with IL-2 increased the number of cells that degranulated in response to targets, but there was still a strong statistically significant inhibition seen with both drugs on degranulation and IFN-γ production (Figure 2A). IL-2 also increased IFN-γ production at low levels of ciclosporin and tacrolimus, but did not reverse the profound inhibition seen even at drug levels corresponding to therapeutic trough levels (Figure 2B). There was no significant change in NK cell numbers with either treatment. These data indicate that NK cell function is likely to be significantly impaired at therapeutic levels of ciclosporin and tacrolimus.

### Loss of NK cell function in the early post transplant period

**Figure 3:**
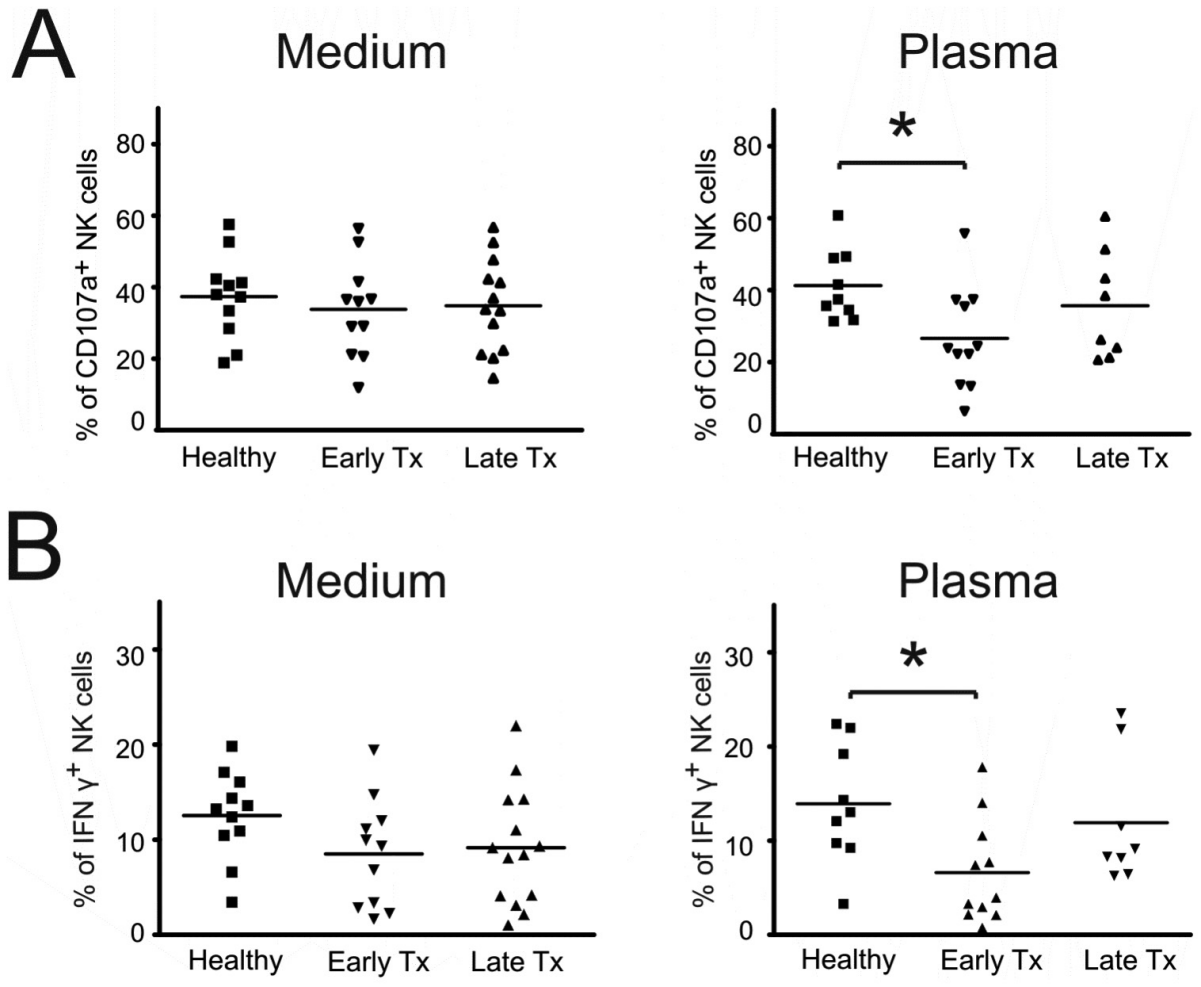
NK cell function is altered in renal transplant patients. (**A**) PBMCs from healthy controls, early transplant patients and late transplant patients were isolated and incubated overnight in culture media or the subject's plasma. NK cell degranulation was assayed the following day by incubation with K562 cells for 6 hrs at a 1∶5 E:T ratio. (**B**) IFN-γ production was evaluated in the same groups as used in (A). Values are individual patients and bars represent means. The data show that when cells are maintained in autologous plasma, NK cells from early transplant patients have impaired NK cell degranulation and IFN-γ production. (* p<0.05 with ANOVA followed by Dunnett's test).

To assess NK cell function in renal transplant patients we compared cells from early (<28 weeks) and late (>4 years) transplant recipients with healthy controls. A cut off of 4 years was used to ensure a wide separation between early and late patients. When NK cells were incubated overnight with targets in medium, no significant differences were seen between the different groups in IFN-γ production or cytotoxic degranulation (Figure 3A left panel and Figure 3B left panel). In the light of our earlier experiments, we considered the possibility that the absence of an effect in these circumstances could be due to the removal of calcineurin antagonists. (The calcineurin antagonist drug levels in these groups are given in Table S3). When PBMCs were incubated overnight in cultures to which the patient's own plasma had been added back, both IFN-γ production and NK cell degranulation were lower in early transplant recipients compared to normal controls (Figure 3A right panel and Figure 3B right panel). Late transplant patients had lower values for both NK degranulation and IFN-γ production than healthy controls, but these changes did not reach statistical significance. When the assays were performed with PBMCs which had been rested in autologous plasma with IL-2, there was an even more marked difference seen in NK cell degranulation between healthy controls and early transplant patients, consistent with a therapy-induced blockade of the effects of IL-2 (54.3±3.1 vs 33.1±4.6%, p<0.05 with ANOVA and Dunnett's test) caused by the drug in the plasma. The addition of IL-2 to cells rested in medium did not increase the discrimination of the assay between the different groups suggesting that the effects seen with plasma are due to drug-induced IL-2 blockade (data not shown). Contingency table analysis using Chi-square testing and Fisher's exact test did not demonstrate any significant difference between the early and late groups in terms of their use of other immunosuppressive drugs (mycophenolate, azathioprine and prednisolone).

### Variation in NK cell numbers in early and late kidney transplant patients

**Figure 4:**
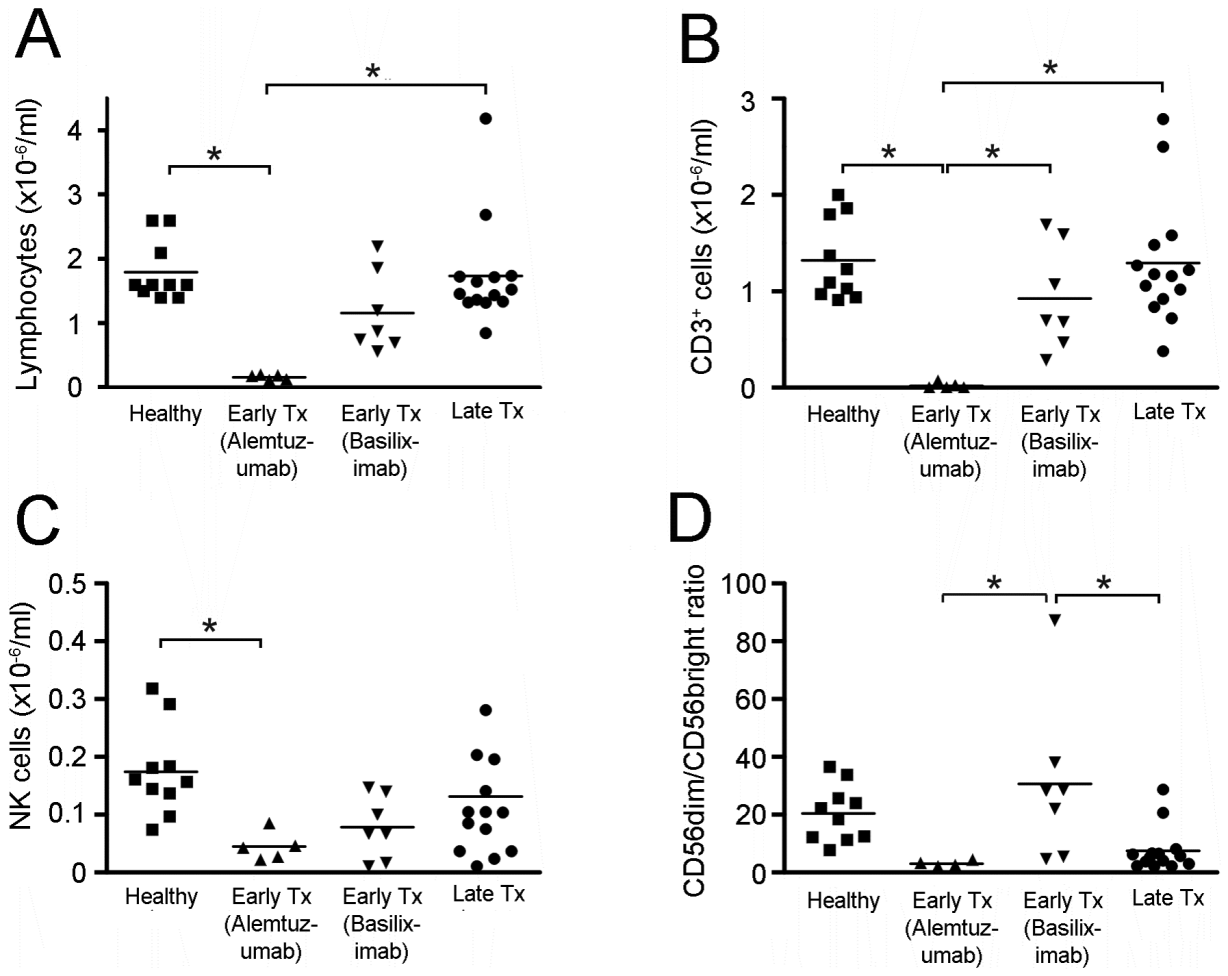
NK cells in peripheral blood cells of renal transplant patients. (**A**) Total lymphocyte counts, (**B**) CD3^+^ lymphocyte counts, (**C**) NK cell counts and (**D**) CD3^−^, CD16^+^, CD56^dim^ : CD56^bright^ NK cell ratios in healthy controls, early transplant patients having received either alemtuzumab or basiliximab, and late transplant patients. The data demonstrate that alemtuzumab treatment is associated with reduced total lymphocyte counts, CD3^+^ lymphocyte counts and NK cell counts. Values are individual patients and bars represent means. (* p<0.05 with ANOVA followed by Dunnett's test or Tukey's test).

To assess the effects of immunosuppression on the development and survival of NK cells, as well as function, we measured absolute numbers of NK cells in blood from patients and controls (Figure 4). For these purposes, early transplant recipients were further subdivided into patients who had received alemtuzumab (anti-CD52) or basiliximab (anti-CD25) therapy because alemtuzumab is known to cause T cell, B cell and dendritic cell depletion and we wished to examine the effects on NK cells. As Figure 4 illustrates, this was informative as there are clearly effects of alemtuzumab on NK cell numbers. No late transplant patients were on alemtuzumab or basiliximab. Compared to healthy controls (1.80±0.15×10^6^ cells/ml), total lymphocyte counts were significantly lower in alemtuzumab-treated early transplant patients (0.16±0.02×10^6^ cells/ml), non-significantly lower in basiliximab-treated early transplant patients (1.17±0.24×10^6^ cells/ml), but preserved in late transplant patients (1.74±0.22×10^6^ cells/ml) (Figure 4A). CD3^+^ T lymphocyte counts were 90-fold lower in alemtuzumab-treated early transplant patients compared to healthy controls (0.02±0.01×10^6^ cells/ml vs 1.80±0.15×10^6^ cells/ml) and non-significantly lower in basiliximab-treated early transplant patients (0.93±0.21×10^6^ cells/ml) and late transplant patients (1.29±0.17×10^6^ cells/ml) (Figure 4B). In contrast, absolute NK cell counts were only 4-fold lower in alemtuzumab-treated early transplant patients compared to healthy controls (0.04±0.01×10^6^ cells/ml vs 0.17±0.02×10^6^ cells/ml) (Figure 4C). Late transplant patients had relative preservation of NK cells (0.13×10^6^±0.03×10^6^ cells/ml). The proportion of NK cells that were CD56^dim^ was non-significantly lower in the alemtuzumab group compared to the healthy controls (Figure 4D). NK cell counts and the ratio of CD56^dim^ to CD56^bright^ NK cells were significantly lower in late transplant patients treated with ciclosporin compared to those treated with tacrolimus) (Table S4). Ciclosporin treatment was also associated with a significant reduction in both NK cell degranulation (29.3±4.0% vs 43.2±4.1%) and IFN-γ production (6.4±1.2% vs. 14.2±3.1%) compared to tacrolimus treatment in this group (Table S4).

## Discussion

In this study we describe the development of a cytometric assay that allows rapid assessment of the two key components of NK cell effector function - cytotoxicity and cytokine secretion. This builds on the ability to detect intracellular IFN-γ production and the repeated demonstration that expression of CD107a correlates closely with NK cell-induced cytotoxic lysis of target cells in Cr^51^-release assays [30], [31], [32]. The flow cytometric assays are robust, reproducible, suitable for a high throughput of clinical samples and can be performed during normal working hours. A wide range of clinical assays exists for B and T cell function and this study addresses the important need for a simple assay of NK cell function that can be used in a variety of disease settings.

An important aspect of the assay is that it does not require pre-separation of individual nucleated blood cell subsets and can be done directly on mixed populations of peripheral blood mononuclear cells after simple counting. The flow cytometric gating used ensures that the assay reports on the proportion of NK cells which have degranulated or are secreting interferon gamma. Unlike a chromium release assay where the measured output is the destruction of the target, this assay uses as its readout the status of the NK cell itself. The output depends on the probability that a given NK cell will encounter a target and then on the probability that this encounter will result in NK effector function. The ratio of 1∶5 for total peripheral blood cells: target cells was used so that there is a very large excess of target cells compared to NK cells, which form only a small proportion of the total peripheral blood mononuclear cell count. This ratio of NK cells to target cells is of the order of 1∶100 and as Figure 1B indicates, this excess is such that there is no further increase in measured effector function with an even higher proportion of target cells in the assay. This indicates that the number of target cells used in the assay is not limiting, presumably because with such an excess of target cells, the probability of an encounter between an NK cell and a target cell is not increased further by the presence of more target cells. Because the number of target cells is not limiting, it is possible to use the assay to assess the probability that an encounter results in NK effector function. As the assay measures the percentage of the NK cells which are activated, this result is not dependent on the absolute number of NK cells in the assay. The output of the assay is relatively stable in individuals over time as indicated by the results in Figure 1C and 1D and the coefficients of variation.

To validate the assays in the context of immunosuppression, we have measured the effects of the calcineurin inhibitors ciclosporin and tacrolimus on NK cell function *in vitro*. Our data demonstrate that both drugs produced dose-dependent inhibition of NK cell degranulation and IFN-γ production *in vitro*. These inhibitory effects were highly statistically significant with strong correlation coefficients. This resolves any uncertainty about the effects of these drugs on human NK cell function. Our findings confirm studies reporting an inhibition of NK cell degranulation with ciclosporin [26], [27], [33] and tacrolimus [33]. Although some reports have suggested that ciclosporin had no effect [24], [25], [34] or slightly increased NK cell activity [28], and that tacrolimus had no effect on NK cell activity [24], [27], we believe these inconsistencies may reflect species-specific differences or the removal of the drug by washing during or prior to the assays [24]. Importantly, the inhibition of NK cell function by ciclosporin is known to be reversible [27] and our *ex vivo* samples required re-addition of autologous plasma, which contained the drug at therapeutic levels, in order to demonstrate difference between cells from patients and healthy controls. The marked reduction in NK cell function in early transplant patients may reflect the higher doses of immunosuppressive drugs which are used in these patients compared to late transplant patients. An enhanced difference was seen when the cells were rested in autologous plasma containing the drug and IL-2. This is consistent with the immunosuppressive drug in the early transplant patients' plasma being responsible for the inhibition of NK cell function. Whilst plasma clearly contains a wide range of biologically active constituents, the evidence indicates that it is likely to be the drug which is causing the effect seen. Consistent with this interpretation, plasma from patients recapitulates the effects seen with drug alone. Furthermore, plasma from normal healthy donors had no effect on the assay results and the addition of IL-2 to cells in medium alone did not alter the discrimination of the assay suggesting that in the absence of the drug IL-2 had similar effects on cells from both healthy donor and transplant patients.

The plasma levels of both ciclosporin and tacrolimus depend on the dose administered and the time between administration and blood sampling. For clinical purposes, drug therapy is usually monitored using the trough level measured before the next dose. However, these are the lowest levels experienced by the patients' immune cells and substantially higher peak levels can be seen [35], [36]. Furthermore, higher drug levels are used in heart, lung and liver transplantation than in kidney transplantation. Our results indicate that both ciclosporin and tacrolimus will have significant NK cell suppressive effects when used as transplant immunosuppression. There are likely to be many factors influencing the appropriate level of transplant immunosuppression including age, co-morbidity, HLA-matching, immunosuppressive drug levels and the function of different immune cell subsets including regulatory T cells, B cells and NK cells. It is likely that the development of any clinical decision-support algorithm to fine tune immunosuppression by altering dosing will have to assess these and potentially other factors. However, the key requirements at present are suitable assays to assess these different variables. We believe that the assay that we have developed will be of great utility for future studies to establish the value of monitoring NK cells function following transplantation. The current study clearly demonstrates that NK cell function is impaired following transplantation and it will be important in future studies to determine the clinical significance of this effect and the value of monitoring it and of fine tuning immunosuppression to minimize this inhibition of NK cell function.

Blood NK cells numbers vary widely in normal populations and therefore we considered that it might be difficult to correlate these changes with functional effects. However, our analysis provides clear evidence that NK cell numbers are relatively preserved in most patients. Alemtuzumab is a known T cell, B cell and dendritic cell depleting antibody [37], [38]. Alemtuzumab has been previously reported to spare NK cells [39], marginally decrease CD16^+^ NK cell counts until 1–2 months after treatment [40], [41], or to deeply suppress NK cells for over 9 months after treatment [42]. Our results demonstrate that compared to other lymphocytes, NK cells are relatively spared by alemtuzumab.

The role of NK cells in viral infection is well established and they play a particular role in the early stages of infection before adaptive immunity can be mobilized [11]. Human NK cell deficiency is associated with severe herpes virus infection [12]. The protection from herpes viruses which is mediated by NK cells is especially relevant to transplantation, where the graft may bring about new viral exposure, especially to cytomegalovirus and there is simultaneous therapeutic suppression of adaptive immunity. Our study indicates that at this critical time, there is also a substantial measurable reduction in NK cell function. Clearly, the optimisation of immunosuppression regimens may require attention to the effects on NK cell function, as excess use of the immunosuppressive regimens that we have studied would lead to profound defects in NK cell function with concomitant risks of disease due to cytomegalovirus and other viruses. The assay that we have developed is sensitive to these effects and can easily be applied to clinical samples.

Natural killer cells play an important role in cancer immunosurveillance and the incidence of cancer is increased following transplantation [43], [44]. In the longer term, patients treated with ciclosporin had lower NK cell counts and weaker NK cell cytotoxic activity than late transplant patients treated with tacrolimus. We also observed a lower CD56^dim^:CD56^bright^ ratio in ciclosporin-treated patients than in tacrolimus-treated patients. Our observations are consistent with a previous study of renal transplant recipients, which showed an early reduction in NK cell numbers with both tacrolimus and ciclosporin with restoration of numbers in the tacrolimus-treated group, but not in the ciclosporin-treated group at one year post transplant [45].

Overall, our results indicate that NK cell numbers and function are strongly influenced by standard immunosuppressive regimens. This is an important finding and is likely to have important consequences in both the early and late post-transplant periods, when viral infections and tumorigenesis can occur. NK cell function is not routinely monitored following transplantation, but our data suggest that it will be important to evaluate NK function in the development of algorithms for tailoring immunosuppression.

## Materials and Methods

### Ethics Statement

This study was approved by the local ethical review committee (Mid and South Buckinghamshire Research Ethics committee; full title: Immune function in autoimmunity and immunosuppression; REC ref number: 08/H0607/50).

### Patient recruitment and clinical samples

Transplant patients from the Oxford Transplant Unit (Oxford Radcliffe Hospitals, Churchill Hospital, Oxford, U.K.) were recruited to the study after giving written informed consent. Healthy controls were also recruited. Late transplant recipients (n = 14) were defined as patients having undergone transplantation at least 4 years prior to blood collection, and early transplant recipients (n = 15) as patients having undergone transplantation 4 to 28 weeks prior to blood collection. A 4 year cut off was chosen to ensure that all late patients were substantially beyond the peri-transplant period. Either ciclosporin or tacrolimus were used as a maintenance treatment in all patients. Induction treatment consisted of two intravenous injections of either 30 mg of alemtuzumab (once post-organ reperfusion and once 24 hrs later), or 20 mg of basiliximab (once before transplantation and once on day 4 post transplantation). Alemtuzumab is a humanized monoclonal antibody against CD52 and causes depletion of cells expressing this antigen. Basiliximab is a humanized monoclonal antibody against CD25 on T cells and blocks effective T cell proliferation. Healthy control samples were obtained from individuals who were well, with no known medical conditions, no inflammatory or infectious conditions and who were not taking any drugs nor had recently used alcohol or smoked. Up to 20 ml of heparinized blood was obtained by venepuncture. When required, this was diluted with twice the volume of phosphate buffer saline (PBS) and centrifuged over 15 ml of Ficoll-Paque Plus for 20 min at 700 g. Mononuclear cells were collected at the interface and washed three times with PBS at 300 g for 10 min. Unless indicated otherwise, the cells were incubated overnight at 37°C in 20 ml of RPMI 1640 medium (supplemented with 10% fetal calf serum (FCS), glutamine and penicillin-streptomycin). PBMCs were counted using a hemocytometer and plated as indicated. For plasma isolation, heparinized blood was centrifuged for 10 min at 300 g and the supernatant stored at 4°C.

### Reagents and cell culture

L-glutamine, penicillin-streptomycin, RPMI 1640, brefeldin A, ciclosporin, and tacrolimus were obtained from Sigma-Aldrich (Poole, U.K.); PBS from HyClone (Cramlington, U.K.); FCS from Biosera (Ringmer, U.K.); bovine serum albumin (BSA) from Fisher-Scientific (Loughborough, U.K.); Ficoll-Paque Plus from GE Healthcare (Piscataway, NJ); IL-2 from Peprotech Ltd (Rocky Hill, NJ); GolgiStop, Cytofix/cytoperm, Permwash II buffer, anti-human CD56-PE-Cy7 and anti-human CD16-PE mouse antibodies from BD Biosciences (San Jose, CA); anti-human CD3-APC-eFluor 780 and anti-human IFN-γ-AlexaFluor 647 antibodies from eBioscience (San Diego, CA); and anti-human CD107a-AlexaFluor 488 from either eBioscience or BioLegend (San Diego, CA). K562 cells were obtained from the American Type Culture Collection and D860 cells were obtained by transfecting Daudi cells with the NKG2D ligand MICA. Where indicated, PBMCs were incubated with various concentrations of ciclosporin or tacrolimus or with IL-2 (100 U/ml) overnight prior to and during the CD107a/IFN-γ assay.

### CD107a (LAMP-1) and intracellular IFN-γ assay

PBMCs were plated in 24-well plates with or without 2.5×10^5^ target cells in a 1 ml final volume of medium, plasma or serum, then incubated, typically for 6 hours at 37°C. After 30 minutes, 0.7 µl GolgiStop (monensin) and anti-human CD107a-AlexaFluor 488 antibody were added to each well. At 6 hours, cells were washed once in 2 ml of PBS, 2% bovine serum albumin, spun for 5 min at 300 g, then stained with anti-human CD3-APC-eFluor780, anti-human CD56-PE-Cy7 and anti-human CD16-PE antibodies for 30 min at room temperature. Cells were washed once in 2 ml of PBS, 2% BSA and resuspended in either 500 µl of PBS, 2% BSA or 500 µl of PBS, 2% paraformaldehyde.

The assay for intracellular IFN-γ was performed in parallel with a few adaptations. After 30 minutes of incubation, the transport inhibitor brefeldin A (0.3 µg/ml) was added to each well along with GolgiStop. After surface staining for CD3, CD56 and CD16, the cells were washed once in PBS, 2% BSA buffer, spun and permeabilized with 100 µl of BD Cytofix/cytoperm buffer, for 20 min at room temperature. The cells were washed twice with 500 µl of BD Perm/Wash buffer II, spun and stained with anti-human IFN-γ-AlexaFluor647 antibody for 30 min at room temperature. Cells were washed once in 2 ml of PBS, 2% BSA and resuspended in 500 µl of PBS, 2% paraformaldehyde. NK cells were identified by gating on CD3^−^ CD56^+^ CD16^+^ lymphocytes.

### Flow cytometry analysis and statistical analysis

Flow cytometry was performed on a BD FACSCanto machine using the BD FACSDiva software. Flow cytometry data were analyzed using the FlowJo 8.8.3 software (Tree Star, Inc., Ashland, OR). Statistical analysis was performed using the Prism 5 software (GraphPad Software, Inc. La Jolla, CA). Two-group comparisons (fig. 1 and 2) were performed using unpaired two-tailed t-tests. Three-group (fig. 3) and four-group (fig. 4) comparisons were done using the one-way ANOVA followed, when significant, by the Tukey's mutiple comparison test or Dunnett's multiple comparison test. Trend analysis was performed using Spearman's test.

## Supporting Information

Table S1 Correlation analysis of CD107a data in Figure 2 using Spearman's test.
(0.03 MB DOC)

Table S2 Correlation analysis of IFN-γ data in Figure 2 using Spearman's test.
(0.03 MB DOC)

Table S3 Drug levels in patient groups.
(0.03 MB DOC)

Table S4 Comparison of results for late transplant patients taking ciclosporin and tacrolimus.
(0.03 MB DOC)

Figure S1 NK cell:Target cell ratio. No correlation was seen between absolute NK cell:Target cell ratio and assay outcome. The outcome of degranulation assays for 27 individuals were analyzed using Pearson's and Spearman's tests for correlation and no significant correlation was seen between the assay outcome and the absolute NK cell count.
(0.06 MB DOC)

## References

[1] Vivier E, Tomasello E, Baratin M, Walzer T, Ugolini S (2008). Functions of natural killer cells.. Nat Immunol.

[2] Lanier LL (2008). Up on the tightrope: natural killer cell activation and inhibition.. Nat Immunol.

[3] Braud VM, Allan DS, O'Callaghan CA, Soderstrom K, D'Andrea A (1998). HLA-E binds to natural killer cell receptors CD94/NKG2A, B and C.. Nature.

[4] O'Callaghan CA, Bell JI (1998). Structure and function of the human MHC class Ib molecules HLA-E, HLA-F and HLA-G.. Immunological Reviews.

[5] Norman PJ, Parham P (2005). Complex interactions: the immunogenetics of human leukocyte antigen and killer cell immunoglobulin-like receptors.. Semin Hematol.

[6] Mistry AR, O'Callaghan CA (2007). Regulation of ligands for the activating receptor NKG2D.. Immunology.

[7] O'Callaghan CA, Cerwenka A, Willcox BE, Lanier LL, Bjorkman PJ (2001). Molecular competition for NKG2D: H60 and RAE1 compete unequally for NKG2D with dominance of H60.. Immunity.

[8] Arnon TI, Markel G, Mandelboim O (2006). Tumor and viral recognition by natural killer cells receptors.. Semin Cancer Biol.

[9] Caligiuri MA (2008). Human natural killer cells.. Blood.

[10] Jacobs R, Hintzen G, Kemper A, Beul K, Kempf S (2001). CD56bright cells differ in their KIR repertoire and cytotoxic features from CD56dim NK cells.. Eur J Immunol.

[11] Welsh RM, Zinkernagel RM (1977). Heterospecific cytotoxic cell activity induced during the first three days of acute lymphocytic choriomeningitis virus infection in mice.. Nature.

[12] Biron CA, Byron KS, Sullivan JL (1989). Severe herpesvirus infections in an adolescent without natural killer cells.. N Engl J Med.

[13] Cosman D, Mullberg J, Sutherland CL, Chin W, Armitage R (2001). ULBPs, Novel MHC class I related moelcules, bind to CMV glycoprotein UL16 and stimulate NK cytotoxicity through the NKG2D receptor.. Immunity.

[14] Hadaya K, de Rham C, Bandelier C, Bandelier C, Ferrari-Lacraz S (2008). Natural killer cell receptor repertoire and their ligands, and the risk of CMV infection after kidney transplantation.. Am J Transplant.

[15] Ruggeri L, Capanni M, Urbani E, Perruccio K, Shlomchik WD (2002). Effectiveness of donor natural killer cell alloreactivity in mismatched hematopoietic transplants.. Science.

[16] Auchincloss H, Sachs DH (1998). Xenogeneic transplantation.. Annu Rev Immunol.

[17] Manilay JO, Sykes M (1998). Natural killer cells and their role in graft rejection.. Curr Opin Immunol.

[18] Blancho G, Buzelin F, Dantal J, Hourmant M, Cantarovich D (1992). Evidence that early acute renal failure may be mediated by CD3- CD16+ cells in a kidney graft recipient with large granular lymphocyte proliferation.. Transplantation.

[19] Kroemer A, Xiao X, Degauque N, Edtinger K, Wei H (2008). The innate NK cells, allograft rejection, and a key role for IL-15.. J Immunol.

[20] Seiler M, Brabcova I, Viklicky O, Hribova P, Rosenberger C (2007). Heightened expression of the cytotoxicity receptor NKG2D correlates with acute and chronic nephropathy after kidney transplantation.. Am J Transplant.

[21] Beilke JN, Kuhl NR, Van Kaer L, Gill RG (2005). NK cells promote islet allograft tolerance via a perforin-dependent mechanism.. Nat Med.

[22] Laffont S, Seillet C, Ortaldo J, Coudert JD, Guery JC (2008). Natural killer cells recruited into lymph nodes inhibit alloreactive T-cell activation through perforin-mediated killing of donor allogeneic dendritic cells.. Blood.

[23] Yu G, Xu X, Vu MD, Kilpatrick ED, Li XC (2006). NK cells promote transplant tolerance by killing donor antigen-presenting cells.. J Exp Med.

[24] Wai LE, Fujiki M, Takeda S, Martinez OM, Krams SM (2008). Rapamycin, but not cyclosporine or FK506, alters natural killer cell function.. Transplantation.

[25] Shao-Hsien C, Lang I, Gunn H, Lydyard P (1983). Effect of in vitro cyclosporin. A treatment on human natural and antibody-dependent cell-mediated cytotoxicity.. Transplantation.

[26] Introna M, Allavena P, Spreafico F, Mantovani A (1981). Inhibition of human natural killer activity by cyclosporin A.. Transplantation.

[27] Wasik M, Gorski A, Stepien-Sopniewska B, Lagodzinski Z (1991). Effect of FK506 versus cyclosporine on human natural and antibody-dependent cytotoxicity reactions in vitro.. Transplantation.

[28] Wang H, Grzywacz B, Sukovich D, McCullar V, Cao Q (2007). The unexpected effect of cyclosporin A on CD56+CD16- and CD56+CD16+ natural killer cell subpopulations.. Blood.

[29] Petersson E, Qi Z, Ekberg H, Ostraat O, Dohlsten M (1997). Activation of alloreactive natural killer cells is resistant to cyclosporine.. Transplantation.

[30] Alter G, Malenfant JM, Altfeld M (2004). CD107a as a functional marker for the identification of natural killer cell activity.. J Immunol Methods.

[31] Aktas E, Kucuksezer UC, Bilgic S, Erten G, Deniz G (2009). Relationship between CD107a expression and cytotoxic activity.. Cell Immunol.

[32] Penack O, Gentilini C, Fischer L, Asemissen AM, Scheibenbogen C (2005). CD56dimCD16neg cells are responsible for natural cytotoxicity against tumor targets.. Leukemia.

[33] Pores-Fernando AT, Gaur S, Doyon MY, Zweifach A (2009). Calcineurin-dependent lytic granule exocytosis in NK-92 natural killer cells.. Cell Immunol.

[34] Lin SJ, Kuo ML (2008). Effect of cyclosporin A on interleukin-15-activated umbilical cord blood natural killer cell function.. Cytotherapy.

[35] Kimikawa M, Kamoya K, Toma H, Teraoka S (2001). Effective oral administration of tacrolimus in renal transplant recipients.. Clin Transplant.

[36] Mahalati K, Lawen J, Kiberd B, Belitsky P (2000). Is 3-hour cyclosporine blood level superior to trough level in early post-renal transplantation period?. J Urol.

[37] Riechmann L, Clark M, Waldmann H, Winter G (1988). Reshaping human antibodies for therapy.. Nature.

[38] Buggins AG, Mufti GJ, Salisbury J, Codd J, Westwood N (2002). Peripheral blood but not tissue dendritic cells express CD52 and are depleted by treatment with alemtuzumab.. Blood.

[39] Isaacs JD, Watts RA, Hazleman BL, Hale G, Keogan MT (1992). Humanised monoclonal antibody therapy for rheumatoid arthritis.. Lancet.

[40] Osterborg A, Werner A, Halapi E, Lundin J, Harmenberg U (1997). Clonal CD8+ and CD52- T cells are induced in responding B cell lymphoma patients treated with Campath-1H (anti-CD52).. Eur J Haematol.

[41] Brett S, Baxter G, Cooper H, Johnston JM, Tite J (1996). Repopulation of blood lymphocyte sub-populations in rheumatoid arthritis patients treated with the depleting humanized monoclonal antibody, CAMPATH-1H.. Immunology.

[42] Lundin J, Porwit-MacDonald A, Rossmann ED, Karlsson C, Edman P (2004). Cellular immune reconstitution after subcutaneous alemtuzumab (anti-CD52 monoclonal antibody, CAMPATH-1H) treatment as first-line therapy for B-cell chronic lymphocytic leukaemia.. Leukemia.

[43] Grulich AE, van Leeuwen MT, Falster MO, Vajdic CM (2007). Incidence of cancers in people with HIV/AIDS compared with immunosuppressed transplant recipients: a meta-analysis.. Lancet.

[44] van Leeuwen MT, Webster AC, McCredie MR, Stewart JH, McDonald SP (2010). Effect of reduced immunosuppression after kidney transplant failure on risk of cancer: population based retrospective cohort study.. BMJ.

[45] Vacher-Coponat H, Brunet C, Moal V, Loundou A, Bonnet E (2006). Tacrolimus/mycophenolate mofetil improved natural killer lymphocyte reconstitution one year after kidney transplant by reference to cyclosporine/azathioprine.. Transplantation.

