# Supplementary material for: Renal Transplant Immunosuppression Impairs Natural Killer Cell Function In Vitro and In Vivo
Source: PLoS One. 2010 Oct 12;5(10):e13294. doi: 10.1371/journal.pone.0013294 (PMC2953494; doi:10.1371/journal.pone.0013294)
Supplement: Table S1 — Correlation analysis of CD107a data in Figure 2 using Spearman's test. (0.03 MB DOC) [file pone.0013294.s001.doc]

**Table S1. Correlation analysis of CD107a data in Figure 2 using Spearman's test.**

| **Drug** | **IL-2** | **Correlation (r_s_)** | **p value** |
| --- | --- | --- | --- |
| Ciclosporin | - | -0.8932 | <0.0001 |
| Ciclosporin | + | -0.673 | 0.0003 |
| Tacrolimus | - | -0.868 | <0.0001 |
| Tacrolimus | + | -0.638 | 0.0005 |
