# Supplementary material for: Renal Transplant Immunosuppression Impairs Natural Killer Cell Function In Vitro and In Vivo
Source: PLoS One. 2010 Oct 12;5(10):e13294. doi: 10.1371/journal.pone.0013294 (PMC2953494; doi:10.1371/journal.pone.0013294)
Supplement: Table S2 — Correlation analysis of IFN-γ data in Figure 2 using Spearman's test. (0.03 MB DOC) [file pone.0013294.s002.doc]

**Table S2. Correlation analysis of IFN-γ data in Figure 2 using Spearman's test.**

| **Drug** | **IL-2** | **Correlation (r_s_)** | **p value** |
| --- | --- | --- | --- |
| Ciclosporin | - | -0.868 | <0.0001 |
| Ciclosporin | + | -0.871 | <0.0001 |
| Tacrolimus | - | -0.865 | <0.0001 |
| Tacrolimus | + | -0.946 | <0.0001 |

r_s_
