# Supplementary material for: Renal Transplant Immunosuppression Impairs Natural Killer Cell Function In Vitro and In Vivo
Source: PLoS One. 2010 Oct 12;5(10):e13294. doi: 10.1371/journal.pone.0013294 (PMC2953494; doi:10.1371/journal.pone.0013294)
Supplement: Table S3 — Drug levels in patient groups. (0.03 MB DOC) [file pone.0013294.s003.doc]

**Table S3. Drug levels in patient groups.**

r_s_

| **Patient Group** | **Drug levels (nM)**  (Mean ± SEM) |
| --- | --- |
| Early Transplantation  (tacrolimus) | 11.7 ± 1.0 |
| Late Transplantation  (ciclosporin) | 61.5 ± 8.3 |
| Late Transplantation  (tacrolimus) | 15.8 ± 3.5 |

Tacrolimus levels did not differ significantly between early and late patients on this drug. No early transplant patients were treated with ciclosporin in line with current protocols.
