# Supplementary material for: Renal Transplant Immunosuppression Impairs Natural Killer Cell Function In Vitro and In Vivo
Source: PLoS One. 2010 Oct 12;5(10):e13294. doi: 10.1371/journal.pone.0013294 (PMC2953494; doi:10.1371/journal.pone.0013294)
Supplement: Table S4 — Comparison of results for late transplant patients taking ciclosporin and tacrolimus. (0.03 MB DOC) [file pone.0013294.s004.doc]

**Table S4. Comparison of results for late transplant patients taking ciclosporin and tacrolimus**

|  | **Ciclosporin**  **(mean ± SEM)** | **Tacrolimus**  **(mean ± SEM)** | **T-test** |
| --- | --- | --- | --- |
| **Lymphocytes (%)** | 1.64 ± 0.14 | 1.91 ± 0.59 | NS |
| **CD3+ cells (%)** | 1.36 ± 0.16 | 1.17 ± 0.42 | NS |
| **NK cells (%)** | 0.06 ± 0.01 | 0.25 ± 0.05 | * P < 0.05 |
| **CD56dim:CD56bright ratio** | 4.18 ± 0.71 | 13.57 ± 4.73 | * P < 0.05 |
| **CD107a^+^ NK cells (%)** | 29.28 ± 4.03 | 43.16 ± 4.13 | * P < 0.05 |
| **IFN-γ^+^ NK cells (%)** | 6.38 ± 1.19 | 14.17 ± 3.11 | * P < 0.05 |
