# Supplementary material for: Renal Transplant Immunosuppression Impairs Natural Killer Cell Function In Vitro and In Vivo
Source: PLoS One. 2010 Oct 12;5(10):e13294. doi: 10.1371/journal.pone.0013294 (PMC2953494; doi:10.1371/journal.pone.0013294)
Supplement: Figure S1 — NK cell:Target cell ratio. No correlation was seen between absolute NK cell:Target cell ratio and assay outcome. The outcome of degranulation assays for 27 individuals were analyzed using Pearson's and Spearman's tests for correlation and no significant correlation was seen between the assay outcome and the absolute NK cell count. (0.06 MB DOC) [file pone.0013294.s005.doc]

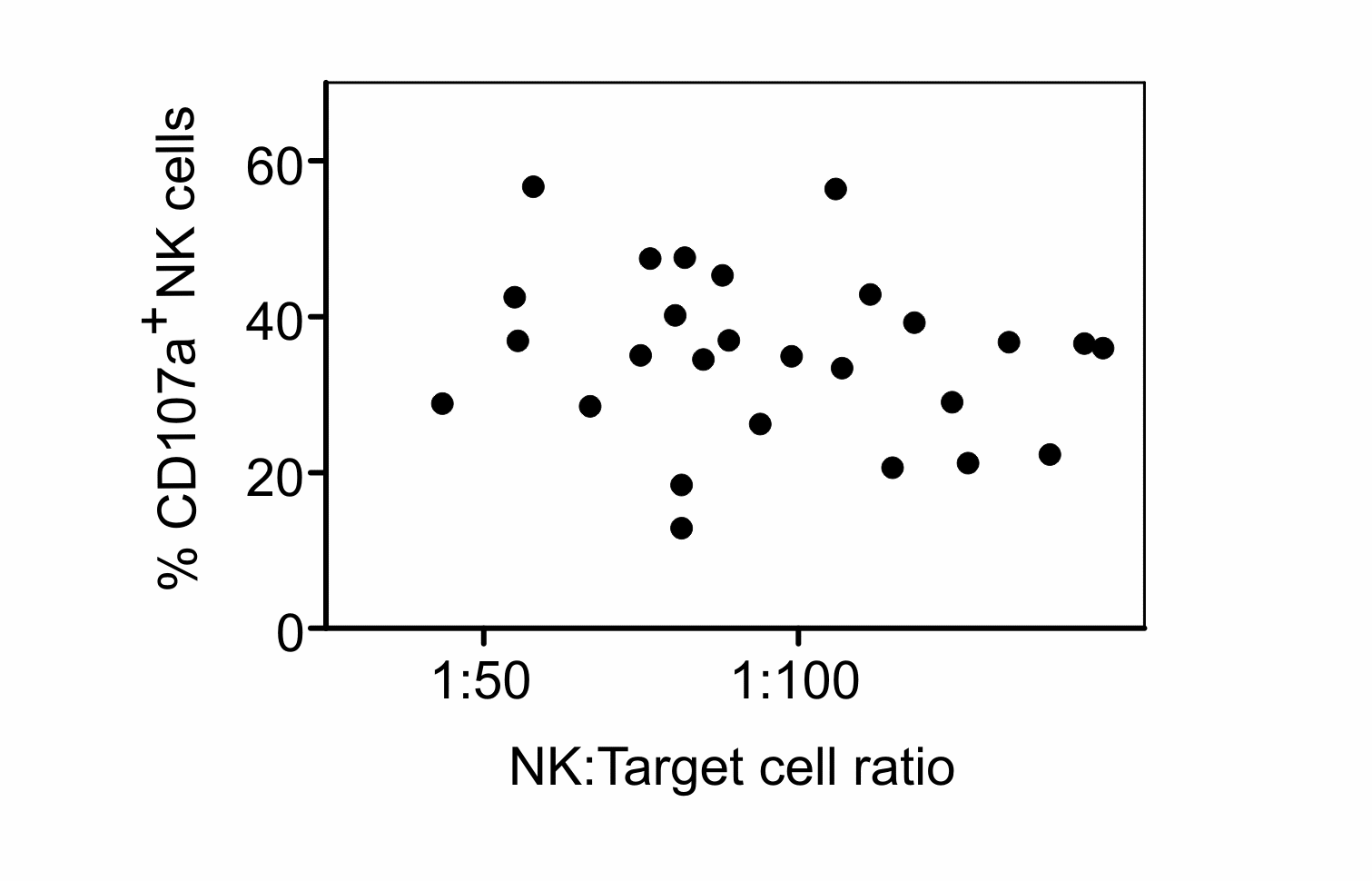


**Figure S1. NK cell:Target cell ratio.**

No correlation was seen between absolute NK cell:Target cell ratio and assay outcome. The outcome of degranulation assays for 27 individuals were analyzed using Pearson's and Spearman's tests for correlation and no significant correlation was seen between the assay outcome and the absolute NK cell count.
